# Employ Your Mind (EYM), a vocational rehabilitation and cognitive remediation program: the participants’ experience

**DOI:** 10.3389/fpsyt.2025.1624213

**Published:** 2025-09-17

**Authors:** Ilona Pruscino, Kirsty Pope, Khalsom Khan Willoughby, Anne Miles

**Affiliations:** ^1^ Department of Occupational Therapy, Monash University, Frankston, VIC, Australia; ^2^ WISE IDEA, WISE Employment, Melbourne, VIC, Australia

**Keywords:** mental health condition, severe mental illness, cognition, cognitive remediation, vocational rehabilitation, recovery

## Abstract

**Background:**

The experience of cognitive challenges due to mental health conditions can significantly affect a person’s level of functioning and recovery including engagement in employment and other vocational pursuits. Cognitive remediation is an evidence-based treatment that has been demonstrated to improve cognitive and functional outcomes when embedded within a psychosocial rehabilitation program. Facilitated by occupational therapists, often with co-facilitators who are mental health workers and/or peer workers, Employ Your Mind is a vocational rehabilitation program incorporating cognitive remediation. To date the lived experience of Employ Your Mind has not been examined. This qualitative study aimed to explore the participant’s perspective of the value of the Employ Your Mind program.

**Methods:**

Semi-structured in-depth interviews were conducted with purposively sampled participants. The qualitative data was transcribed verbatim prior to undergoing thematic analysis. The utilisation of member checking and researcher reflexivity augmented the trustworthiness of this study.

**Results:**

Findings from participants (n=4) highlights the requirement for a ‘just-right challenge’, heightens the value of the therapeutic relationship and reinforces the advantages of flexible service provision to harness participant motivation and promote optimal outcomes.

**Discussion:**

This study reinforces the necessity of obtaining the lived experiences of participants to shape the evolution of cognitive remediation, particularly in the field of vocational rehabilitation, by providing a qualitative voice to enhance program delivery.

## 1 Introduction

The World Health Organisation (WHO) reports that one in every eight people in the world live with a mental health condition, with more significant conditions such as Schizophrenia affecting approximately 24 million people or one in 300 people worldwide (2). The term mental health condition can be used to describe a range of, diagnoses a person may experience including schizophrenia, depression, and bipolar disorder (3). Many people who live with such conditions can experience cognitive and functional challenges including difficulties with concentration, planning, and working memory (4, 5). These cognitive skills are necessary to support engagement in competitive employment opportunities (6). Mental health conditions, therefore, can affect participation in employment and the fulfilment of vocational goals (5, 7) which form a valuable component of mental health recovery (8). Despite a strong desire to work (9) and the significant benefits that can be derived from employment (10), people with schizophrenia experience low rates of employment, with one large scale international study reporting employment rates for this group ranging from 16% in the East Asia region to 23% in Northern Europe (11). These low rates of employment are of critical concern considering exclusion from these meaningful occupations negatively affects the health and wellbeing of individuals living with mental health conditions, contributing to social isolation, financial insecurity, and increased anxiety (12, 13). This is illustrative of the bidirectional relationship between mental health and unemployment (14) and the impact that living with a mental health condition has upon job tenure (5).

Cognitive remediation (CR) has been identified as an effective intervention to enhance cognitive and functional abilities in order to improve vocational outcomes (15). CR is defined as ‘an intervention targeting cognition (attention, memory, executive function, social cognition, and meta-cognition) using scientific principles of learning with the ultimate goal of improving functional outcomes. Its effectiveness is enhanced when provided in context (formal or informal) that provides support and opportunity for extending to everyday functioning’ (16). The origins of CR date back to the nineties, initially focused on people with a diagnosis of schizophrenia (17). Since then, CR has substantially evolved into an evidence base for a range of mental health conditions (18).

Facilitation of CR is typically provided by an array of health professions including but not limited to, psychologists, occupational therapists (OTs), nurses and social workers, with therapist training primarily occurring within the practical context (19). While the authors are not aware of published research to date specifically regarding the role of OT in cognitive remediation, there are several references to OTs being involved in, or recommend the provision of, CR delivery in the literature (20–25). The utilisation of CR by OTs is also referenced in OT Australia’s Mental Health Occupational Therapy Capability Framework (26). With an emphasis on functional goals and ‘real world’ improvements, CR can be considered as within the scope of practice for occupational therapists.

CR targets cognitive domains, building on the tenets of neuroplasticity (27) to drive generalised advancements in functioning (6, 28). Within vocational rehabilitation, this is necessary to improve problem-solving, social abilities and communication, all essential skills for employment (29). Four core components of CR have been identified to induce positive therapeutic outcomes: facilitation by a trained CR therapist; cognitive exercise; procedures to develop problem-solving and; transfer to real-world scenarios (18).

CR has been established as an intervention that can be effectively utilised within vocational rehabilitation programs (6, 30) with improvements in cognitive and functional domains, including attention, executive functioning, and working memory identified in the literature (6, 27). Findings also support the utilisation of CR to produce improvements to three vocational domains including paid employment, volunteering, and further education (31). Engagement in vocational pursuits following CR is associated with improved self-efficacy, employability, and quality of life; and is, therefore, an essential activity to for people with mental health conditions to access (21, 28, 29, 31, 32).

Two examples of vocational rehabilitation programs with embedded CR include Thinking Skills for Work (33) and the more recently developed Minds@Work (34). Thinking Skills for Work was first developed in the early 2000’s specifically to address the barriers to employment as a result of cognitive difficulties associated with mental health conditions. Multiple randomised controlled trials have been published that validate its effectiveness, but to our knowledge, there has been no in-depth exploration of the participant’s experience. Minds@Work, developed specifically to include components to harness the predictors of job tenure, has also demonstrated preliminary feasibility and acceptability, with further validation studies recommended by the authors (35).

The current study was undertaken in Melbourne, Australia, alongside WISE Employment’s WISE Ways to Work (WWtW) program. WWtW was established to assist people with mental health conditions who have vocational ambitions including goals for employment, volunteering and study. After the completion of this study, WWtW’s name changed to WISE IDEA (Innovation, Development, Evaluation, Application), a Centre for Best Practice in Mental Health and Employment. A key goal for WISE IDEA is to address many of the obstacles faced by people with mental health conditions by providing evidence-based vocational supports, whilst concurrently assisting employers to provide a safe and responsive work environment. This is in line with recommendations from the Royal Commission into Victoria’s Mental Health System, which identifies the need for ‘mentally healthy workplaces’ by promoting inclusion, fostering safe environments, and supporting those with mental health conditions to engage safely in paid employment opportunities (36).

One of the vocational rehabilitation interventions offered by WISE IDEA is the Employ Your Mind (EYM) program, which effectively incorporates CR throughout delivery. WISE IDEA is the only provider of EYM in Australia, having adapted the program from Fife Employment Access Trust in Scotland (37). EYM encompasses four phases, spanning six months, and incorporates group-based learning/discussions and computer-based CR (**Table 1**). In Australia, EYM has been facilitated by a combination of Master’s qualified OTs and community mental health workers from a range of backgrounds, including peer work. Peer workers are members of the Lived Experience workforce and have ‘personal experience of mental health challenges, service use, periods of healing/personal recovery’ (38). The EYM program is designed to improve the cognitive and functional capacities of individuals living with mental health conditions, including their ability to engage in social interactions, plan, and set meaningful vocational goals (39).

**Table 1:** Outline of EYM program phases and components.

Phase	Delivery and duration	Components
Phase 1	Individual: one 90-minute session per week for 6 weeks	Introduction to cognition, cognitive remediation and EYM program. Completion of cognitive and psychosocial exploration. Completion of strength-based character survey. Interest exploration and goal setting.
Phase 2	Group: two 90-minute sessions per week for 6 weeks	Exploration of communication, social cognition, and goal setting in context of work/vocation. Commencement of individual project work. Thinking Gym – on-line CR delivery using HappyNeuron Pro. End of Phase group celebration and certificate.
Phase 3	Group: two 90-minute sessions per week for 6 weeks	Exploration of communication, social cognition, problem solving, physical health, cognitive health and goal setting in context of work/vocation. Completion of individual project work and presentation. Thinking Gym – on-line CR delivery using HappyNeuron Pro. End of Phase group celebration and certificate.
Phase 4	Group and Individual: two 90-minute sessions per week for 6 weeks	Option for up to 5 volunteer work experience sessions (< 20hrs). Thinking Gym – on-line CR delivery using HappyNeuron Pro. Completion of cognitive and psychosocial exploration including progress discussion and report. Graduation group celebration and certificate.

CR service provision can be enhanced through the application of Self-Determination Theory (SDT) to harness participant motivation and support learning (40). SDT proposes that individuals are motivated to learn and develop to fulfil three innate needs: autonomy, relatedness, and competency (41). SDT proposes that individuals are motivated to learn and develop to fulfil three innate needs: autonomy, relatedness, and competency (40). This theory underpins one’s rationale for engagement in activities of daily living and forms a necessary consideration throughout CR, to promote optimal conditions for growth and independence (40). EYM is underpinned by SDT by emphasising autonomy, competence, and relatedness to enhance motivation and engagement. For example, in order to foster autonomy, participants are supported to set goals that are meaningful to them and are encouraged to reflect on their progress, identify areas of strength and growth, and celebrate their achievements. In addition, participants choose their own personal project to work on over the course of the EYM program. To promote competence, computer-based activities are scaffolded to match participants’ skill levels, allowing them to experience success and build confidence in their cognitive abilities. To cultivate a sense of relatedness to enhance motivation and engagement, participants are encouraged to engage in discussions with fellow group members, share experiences, and develop trusting therapeutic relationships with group facilitators including peer worker co-facilitators who themselves have lived experience of mental health conditions.

In this setting, quantitative data mirrors the CR literature in demonstrating the efficacy of EYM with improvements demonstrated in objective and subjective cognition, quality of life, wellbeing and self-perceived working ability (21, 39). To date, however, few studies have examined the lived experience of engagement within CR, and to our knowledge, there has been no in-depth exploration of the participant experience of vocationally focused CR programs. As part of the Minds@Work study (35), short questionnaires were utilised to measure participant satisfaction. The participant experience of Thinking Skills for Work has been reflected in case studies (33, 42) but with a strong focus on quantitative study design, further in-depth analysis of the subjective experience does not appear to have been undertaken to date.

Of the studies that have conducted qualitative exploration of non-vocationally focussed CR approaches, both positive experiences and areas for improvement are reported. Positive respondent perspectives share the importance of the therapist and indicated that CR resulted in improved cognition, social connection, self-efficacy, autonomy, use of cognitive strategies, and emphasized participants’ increased capacity to transfer learnings to real-world scenarios leading to functional improvements (32, 43–46). Participants of CR programs also identified important areas for consideration including the ‘costs’ of CR involving psychological costs such as performance anxiety and frustration, and practical costs including the length/duration of the intervention (32, 43, 45). These factors are important to consider when facilitating CR programs, in addition to some participants reporting a lack of transfer of skills into their everyday lives (43, 47). More recently, a lived experience account of a cognitive remediation therapy program (CIRCuiTS), facilitated by OTs and allied mental health workers, identified the importance of the strengths based approach, monitoring of personal goals, sharing of success, development of cognitive strategies and metacognition in order to improve cognitive abilities and experience real world improvements (22). Obtaining consumer perspectives is important in highlighting enablers and constraints to engagement, providing critical insights to ensure cognitive remediation continues to evolve with the needs of those service users (48, 49).

A detailed understanding of the participant’s lived experience is therefore integral to inform enhancements to EYM curricula and delivery. This study serves to build upon previous analyses (21), to enrich the understanding of EYM and its acceptability from the participant perspective.

**Aim:** The aim of the study was to explore the participants’ perspectives of the value of EYM.

### 1.1 Research questions

1. What are the opinions and perspectives of participants in the delivery and content of EYM?
2. What are the participants’ perspectives of the impact of EYM on their vocational goals?

## 2 Materials and methods

A qualitative methodology was utilised to gather subjective data. Such an approach is concentrated on procuring verbatim data, to analyse and understand human experiences (50). A constructivist paradigm was also adopted as this provides the foundation of qualitative research in rejecting the notion of one objective reality and promoting the gathering of subjective lived experiences (51). This study was approved by the Monash University Human Research Ethics Committee (Approval number 30805). A consolidated criteria for reporting qualitative research (COREQ) (52) was completed prior to submission to ensure reporting important aspects of this research.

### 2.1 Sampling and recruitment

Purposive sampling was applied to enlist participants between February and May 2022. In addition to the existing EYM program eligibility criteria of being over the age of 18, having a diagnosed mental health condition, a basic level of English literacy and a vocational goal, any participant who had completed at least Phase 2 of EYM within the previous 18-months was eligible (n=17) to take part in the research. Those who met the eligibility criteria and opted in were therefore included in the study.

Throughout the recruitment process, vulnerabilities of this population were recognised, and, therefore, to mitigate any power imbalances, a third-party was sourced to distribute recruitment emails. This sought to preserve autonomy and nullify the risk of coercion, by enabling participants to have complete control over their decision to participate in the study, despite no monetary incentive, nor any direct benefits to them (53). The third-party sent potential participants an email invitation which included an explanatory research statement detailing the purpose of the research. Four participants responded to the email invitation and subsequently consented to engage in the research. Informed written consent was obtained prior to engagement and verbal consent prior to audio recording of the interviews.

### 2.2 Data collection

Qualitative data was obtained through semi-structured, in-depth interviews conducted by the first author with the respondent. The interview guide was constructed by the research team, with input from the second, third and fourth authors who share extensive experience within the mental health and vocational rehabilitation sectors. Limitations from prior qualitative papers examining CR were acknowledged, resulting in the inclusion of increased open-ended questions to acquire descriptive accounts, whilst upholding consistency across all interviews.

Interviews were conducted via video conferencing or telephone, in accordance with participant preferences. This sought to reduce the participant burden by increasing participant autonomy and control (54). The questions were open-ended with neutral probes employed. Neutral probes were utilised to avoid confirmation bias and provide participants with the opportunity to expand on their responses (55). The interviews ranged from 30-60 minutes and were audio recorded. Data was then transcribed verbatim with pseudonyms allocated to maintain confidentiality. A complete transcript was returned to participants for member checking, thus ensuring credibility of findings (56). No changes to the transcripts were suggested by the participants. All electronic data was transferred to LabArchives, an electronic laboratory notebook (ELN). The ELN is password protected, only accessible by the first and second authors.

### 2.3 Data analysis

Data analysis procedures were informed by Braun and Clarke’s (1) approach to thematic analysis (**Table 2**). An inductive analysis was undertaken to ensure that findings were derived from the data and representative of the subjective responses (57). Researcher reflexivity included the first author compiling an audit trail to aid reproducibility (57), and writing written reflections immediately following project occurrences. Interpretation bias was reduced through the first author not having been involved in the delivery of the EYM program and therefore having limited pre-conceived views when analysing the data.

**Table 2:** Thematic analysis procedures.

Stage	Example
1. Familiarising yourself with the dataset	The first author initially familiarised herself with the data by transcribing the interviews verbatim into Word documents prior to reading and re-reading the interview transcripts.
2. Coding	Following data familiarisation, the first author completed marginal notes next to each line. Then common elements were highlighted by both the first and second author independently. Initial codes were then noted next to each line.
3. Generating initial themes	The first and second author then examined the codes independently, adopting an iterative process to generate initial themes which appropriately reflected the codes. Both authors then came to a consensus.
4. Developing and reviewing themes	Both authors then re-examined the transcripts to ensure that the themes decided upon were an accurate reflection of the lived experiences provided by respondents.
5. Refining, defining and naming themes	The themes were then further refined by allocating names and defining them in relation to the data they represented
6. Writing up	The first author then constructed the data analysis report, postulating the relationship between the themes and two research questions. Participant quotations were then selected to further accentuate key findings and ensure authenticity of participant responses.

## 3 Results

Demographic data concerning age, gender and mode of program delivery, was collected from all participants (n=4) (**Table 3**). The sample consisted of two people who identify as male and two people who identify as female, all of whom had completed EYM, across different modalities including face-to-face and on-line remote delivery. Participants’ diagnoses were collected separately, with primary diagnoses consisting of Schizophrenia, Bipolar Disorder and Level 1 Autism Spectrum Disorder, and secondary diagnoses including anxiety and depression. While this sample is small, it is representative of the gender distribution and average age of the participants that took part in two previous evaluations of EYM (21, 39).

**Table 3:** Demographic information.

Pseudonym	Age	Gender	Mode of Program Delivery	Vocational Status at EYM commencement
Amanda	42-47	F	Online (One-to-One)	Employed
Bob	42-47	M	Online and Face-to-Face (Group)	Volunteering
Charlotte	54-59	F	Online (Group)	Unemployed
Dan	24-29	M	Online and Face-to-Face (Group)	Unemployed – Not looking for work

Thematic analysis identified five salient themes: mode of delivery, therapeutic relationship, program content, cognitive and functional advancements, and vocational outcomes.

### 3.1 Mode of delivery

The alternative ways that the program was delivered to ensure its continuation throughout the COVID 19 pandemic were evident throughout participants’ nuanced descriptions. Each respondent had varied experiences, with some participants completing the program entirely online, whilst others had a combination of online and face-to-face engagement (**Table 3**). For Bob, the ability to engage online provided a new learning experience, “*I’m getting some computer skills going*”. This enabled him to navigate the online world and grasp new computer skills “*outside [his] comfort zone*”, whilst also providing an outlet for social interactions during stringent lockdowns. Similarly, Charlotte reported the advantages of online engagement, including the establishment of routines during the pandemic whilst providing “*something to look forward to*” quoting, “*it really was the highlight of my week*”. However, despite reporting advantageous aspects of online participation, both participants argued that a face-to-face facilitation “*would have been better*” (Bob). Charlotte proposed that a face-to-face group-based facilitation would have increased opportunities for social interactions, stating “*we could have made … better relationships*” as “*there wasn’t really the opportunity, unfortunately*”. Describing the difficulty in establishing those attachments online, she reported “*it’s hard to … build a relationship*”. Dan, who completed the majority of the program in-person, primarily attributed his enjoyment of the program to the face-to-face sessions, stating that this environment enabled him to contribute “*more than I ever have*” as it provided a safe space where he could develop a rapport and “*socialise with the workers*”.

Contrastingly, after attending three group sessions, Amanda transitioned to complete the program online via a one-to-one facilitation. This enabled for continual engagement in paid employment throughout the entirety of the program. She commented that, had this modality of service provision not been available, she would have “*pulled out of the program*” as it would not have been financially viable for her to miss work in order to attend sessions. Furthermore, group environments were evaluated by Amanda, as “*triggering*”, to which she drew parallels to her experience with group settings in psychiatric hospitals, commenting that she found this environment “*confronting*” and laden with “*emotional connotations*”. Amanda reported, “*being able to do it a bit more privately has allowed me to actually tell them … what was actually on my mind*”. Contrastingly, Charlotte testified she “*wouldn’t have liked to have done one-on-one*”, as she valued the social opportunities, stating, “*I enjoyed the group aspect of it*”.

### 3.2 Therapeutic relationship

The therapeutic relationship was acknowledged by all, with positive accounts, when referring to the facilitators. The proficiency of the facilitators was unanimously reported by participants, describing them as “*knowledgeable … approachable … and solution-oriented*” (Amanda), whilst providing an appropriate “*level of guidance*” (Amanda). Participants collectively expressed the positive influence the “*very capable coaches*” (Bob) had on their program participation, stating, “*they are the ones who made the sessions come alive*” (Charlotte). Furthermore, the facilitators elicited feelings of safety and security amongst participants. Dan described them as “*very supportive*” and highlighted their capacity to create a space where everyone was welcome to participate, “*I actually contributed more than I ever did*”. Amanda also acknowledged the role of the therapeutic relationship, divulging that the facilitator in the one-on-one environment “*kind of held my hand*”, and “*made me feel safe and supported to be vulnerable*”.

Additionally, two of the participants referred to the healing capacity of the therapeutic alliance. Amanda reported that her engagement in EYM “*helped [her] to heal*”, highlighting the advantageous influence of the facilitators, vocalising that “*there’s been healing and acceptance in myself”* and a “*shift in my identity*”. She described their influence in “*helping [her] understand*” the influence of her diagnosis upon engagement. She stated, “*I was blown away … that kind of explains so much*”, by breaking down “*the different components … within [her] presentation*”. Similarly, Charlotte contended the positive influence of the program stating, “*[it] helped me to get better, helped my mind get better*”, commenting that “*it was holistic*” with a broad emphasis on wellbeing with “*a focus on food and health and exercise*” (Charlotte).

### 3.3 Content

Diverse perspectives were provided concerning EYM’s content. In presenting the experiences of participants, the application of the ‘Goldilocks Analogy’ assists in comprehending the differing lived experiences. For Amanda, the content was perceived as “a *little bit … basic*”, whereas for Bob and Charlotte, the content was described as a “*challenge*”. For Dan, the “*difficulty was just right*”. Despite varied accounts, three out of four participants still viewed the content positively. Although basic, Amanda found the curriculum “*really relevant*” in enabling her to “*build … capacities*” through a personalised approach. Likewise, Bob and Charlotte described the curriculum as a “*good challenge*” for “*where my mind was at*” (Charlotte), with cognitive exercises “*pitched at a good level*” (Bob), whilst also being “*fun and engaging*” (Bob). Notedly, despite Dan saying that it “*was just right*”, he also reported “*it got a bit old after a while*” and that he “*didn’t like that it was mostly the same throughout*”, suggesting that “*they could have tried something different*”.

However, when appraising the cognitive remediation (CR) content of EYM, participants unanimously affirmed positive experiences. These accounts ranged from “*I think it was pretty good*” (Bob) to “*I loved it!*” (Amanda). Participants commented on the impact CR had upon their everyday life, describing improvements “*in terms of actually solving a problem*” (Dan), developing social skills to “*build interpersonal … relationships*”, and increasing independence, “t*he CR did help with my independence*” (Dan). Additionally, the computer-based facilitation of CR using the on-line platform HappyNeuron Pro, colloquially termed the ‘Thinking Gym’, was positively received by all. Participants described the “*good variety of games*” (Bob) available to them, “*the games were brilliant*” (Charlotte).

The “*strengths-based*” (Amanda) approach to EYM was acknowledged by all. Charlotte recognised that EYM “*encouraged you to acknowledge your strengths … and appreciate them*”. Similarly, Amanda purported that EYM is “*not a psychiatric kind of model*” rather it is a “*strength-capacity building approach*” concentrating on “*what you can do, not what you won’t ever be able to do because of your … illness*”. Amanda went on to describe that having a mental health condition previously meant that she had “*always been looked at from a deficit point of view*” and that EYM instead provided an opportunity to “*manage those deficits in my skills*” and presented as an “*opportunity to actually change people’s lives*”. Amanda also supported its application within a preventative capacity, to “*support people who are in employment to stay in employment*”, postulating that EYM and its strengths-based focus was a “*better process for me than any psychology I’ve ever done*”, reporting that she “*would love to see other people have the opportunity to do it [EYM]*”. Correspondingly, Bob and Charlotte provided supportive statements, enunciating that “[*EYM] was worthwhile*” (Bob), and that “*I cannot express enough how much I enjoyed it*” (Charlotte).

### 3.4 Cognitive and functional outcomes

All participants reported improvements to cognitive and functional domains including concentration, social capacities, problem-solving, and executive functioning. Amanda articulated instances where the cognitive exercises had improved her concentration within her paid work role by providing “*a good structure*” to ensure that the “*skills are transferable*”. Both Bob and Dan reported that CR “*positively*” (Bob) influenced communication and problem-solving stating, “*I can solve problems easier*” (Dan). Upon reflection participants identified real-world scenarios where these advancements assisted them, including in social situations, “*I’ve become more aware*”, and when volunteering, “*I’ve improved my voluntary work*” (Bob). In addition to an increase of paid working hours, Amanda articulated that EYM enabled her to develop skills specific to her paid-work role, including concentration and executive functioning, and subsequently, “*work … on those challenges that are real in my life*”, providing her with the opportunity to “*improve my skills in employment*”.

### 3.5 Vocational outcomes

At the time of interviewing, Amanda had continued her paid role, with increased hours and responsibility; Charlotte, after a period of unemployment, returned to a paid position in the same field; Bob had maintained engagement in volunteering, and Dan did not have plans to pursue any form of vocational engagement in the immediate future.

Amanda commented on the capacity of EYM to build on skills specific to her paid role enabling her to “*manage those* sp*ecific deficits in my skills from my exact scenario rather than a generalised kind of thing, I think that’s been brilliant*”. Similarly, Charlotte acknowledged that she felt as though “*indirectly … it definitely helped me get better, helped my mind get better*”. Bob reported that “*I don’t have a job but … I’m closer to getting a job*”. He emphasised that EYM strengthened his capacities to engage in voluntary pursuits, “*I’ve learnt new skills*” and “*improved my voluntary work*”.

## 4 Discussion

This study sought to explore the participants’ perspective of completing a vocational rehabilitation and cognitive remediation (CR) program, EYM. To our knowledge, it provides the first qualitative review of this type of program. The application of the ‘Goldilocks Analogy’ highlights varied lived experiences, and that the content and facilitation of EYM is not a ‘one-size-fits all’ approach. Instead, a range of factors must be considered, including person-centred care, the therapeutic relationship, mode of program delivery, and a ‘just-right challenge’ to ensure optimal outcomes.

The World Health Organisation (58) postulates the necessity of person-centred practice throughout mental health service provision to ensure continuity of care and better health outcomes. Ultimately, this approach seeks to ensure a personalised service whereby the participant is viewed as the expert of their own needs, and the therapeutic relationship is guiding rather than directive (59) this is further reflective of both the recovery model and self-determination theory, necessitating participant autonomy to improve outcomes for individuals living with mental health conditions (40). Within EYM, this is particularly operationalised in Phase 1 (see **Table 1**), whereby participants engage independently with the facilitators. This enables the development of rapport, the exploration of strengths and challenges and setting individualised goals, whilst empowering participants to judge whether EYM will be a suitable program for them, prior to exposing them to a group environment. A person-centric lens is therefore essential to provide the participant with increased autonomy throughout their rehabilitation journey (59).

This study highlights that participants valued the therapeutic relationship and appraised the facilitators as pivotal to their engagement, unanimously agreeing that they were integral to the program’s success. These findings support the significance of lived experience qualitative data as the value and mechanism of this relationship is not evident in quantitative measures. This qualitative study therefore adds to the limited, but growing breadth of, literature, reinforcing the benefits of the therapeutic relationship throughout cognitive remediation from the participant perspective (22, 44, 45, 47). As such, it is necessary to acknowledge factors that may impede the development of rapport and subsequent positive participant experiences within vocational rehabilitation and cognitive remediation programs.

The findings of this study illustrate advantages and drawbacks of online and face-to-face service provision. Previous research has highlighted difficulties in the establishment of a therapeutic alliance during the early stages of rapport-building in an online environment (60, 61). Cella et al. (62) found that the online delivery of CR was well-received and could potentially become a standard practice, provided that factors such as digital proficiency, access to necessary technology, internet connectivity and therapists’ abilities are considered. Moreover, the study found that online CR sessions resulted in heightened participant engagement and reduced dropout rates compared to in-person delivery sessions (30). Participants also expressed satisfaction with the online format, reporting cognitive enhancements and practical benefits for their daily routines. These benefits were attributed to the flexibility in session scheduling, the elimination of commuting requirements, and the therapists’ adeptness in fostering participant engagement and cultivating positive therapeutic relationships. Research underscores the significance of such relationships for enhancing engagement (63). These findings align with Amanda’s experience. As such, based on the findings and Amanda’s perspective, it is important to provide the flexibility of online delivery when suitable, particularly when accommodating the schedules of those already engaged in vocational activity such as paid employment.

Whilst a person-centric approach is essential to promote autonomy and ensure continuity of care, clinical reasoning is still required from the facilitators to acknowledge what environments would create the best outcomes for each participant. Literature to date supports group-based, face-to-face service provision, corroborating its capacity to promote the development of rapport and improve employability attributes (17, 64). However, a more recent study provided varied perspectives (61). Therefore, further research is warranted to detail the most efficacious mode of service provision for individuals experiencing mental health conditions, with flexibility of approach and modality potentially useful when looking to benefit those not able to access face-to face services due to location or circumstance.

The findings of this study illustrate the need for a personalised approach to cognitive exercises to ensure a ‘just-right challenge’. This concept, central to occupational therapy, refers to creating an appropriate challenge for an individual’s unique skill set, confirming that the level of difficulty is suited for optimal learning (65). If a task is perceived as too easy, it fosters disengagement and avolition. However, if a task is perceived as too challenging, it can lead to a reduced self-efficacy and subsequent disengagement (65). Thus, ascertaining a just-right challenge is necessary to motivate and challenge a person within appropriate limits (65). This construct is evident within EYM’s ‘Thinking Gym’, which utilises the HappyNeuron Pro on-line platform, titrated to match participant’s responses. Ensuring appropriate levels of stimulation, seeks to build on the tenets of neuroplasticity and produce generalised advancements (28). It is, therefore, necessary to generalise the just-right challenge throughout cognitive remediation to ensure a graded learning environment (18, 40). Concurrent with the core components required for CR, this method seeks to endorse a graded difficulty of cognitive exercise to provide appropriate stimulation, improve occupational engagement and negate the influence of avolition (18, 40). Endorsing a just-right challenge, therefore harnesses SDT to motivate participants to engage in an approach that is suited to their learning capacities, ultimately, promoting independence, social connectedness and proficiency (40).

Participants reported improvements to cognitive and functional outcomes, with vocational outcomes including increases in job responsibility, hours worked, re-employment and continuation of volunteer work with future plans to pursue paid employment described by three out of four of the participants interviewed. These findings are consistent with historical analyses of CR (6, 27), and preliminary quantitative analyses of EYM (21, 39), thus, reinforcing the efficacy of EYM to elicit improvements in psychosocial and cognitive domains.

### 4.1 Implications

Although sample size was limited, this qualitative study provides important insight to the participants’ experience of a vocational rehabilitation program with embedded CR. The findings reflect those of previous qualitative studies, adding to the depth of literature in this relatively under explored area. It also provides insight into the subjective experiences of those interested in fulfilling their vocational goals and the importance of the flexible, strength based, recovery focussed approach in order to do so.

The findings are primarily of interest to those working within CR program design and delivery, particularly in vocational rehabilitation and mental health sectors. The following recommendations can contribute to the continual improvement of CR for individuals with goals for employment, study or volunteering:

- Adoption of a strengths-based person-centric lens throughout cognitive remediation to enhance intrinsic motivation.
- Provision of a flexible mode of delivery (particularly to accommodate working hours) with opportunities to build rapport and develop skills for vocational engagement, with further research into the efficacy of on-line delivery of EYM and other CR interventions.
- Recognition of the importance of the therapeutic relationship, and positive interactions between participants provided by the group context, to provide a supported learning environment.
- A graded approach to cognitive exercises to ensure a ‘just-right challenge’.
- Clear explanation and reinforcement of the need for ‘repeated practice’ in order to understand the repetitive nature of some cognitive exercises.
- A comprehensive understanding of SDT, to harness participant volition concurrent with improved learning outcomes.

### 4.2 Limitations

Limitations within this study warrant acknowledgement. Difficulties with recruitment resulted in a limited sample (n=4) from a subject pool of 17 participants to recruit from. A range of factors impacted recruitment, these included the length of time between potential participants completing EYM and being invited to engage in the study, the influence of ongoing COVID restrictions in Melbourne, and the difficulty re-engaging potential participants with EYM after having completed and left the program. This constrained sample resulted in the inability to generalise findings to broader populations whilst impeding the potential for sufficient analyses. Moreover, a limited sample with constrained stratification did not allow for detailed analyses. A larger sample and longitudinal data collection would be required to establish the relationship between EYM and vocational outcomes. Due to project scope constraints, this research did not include co-design principles whereby participants were engaged throughout the construction of the research proposal. Consistent with the Royal Commission into Victoria’s Mental Health System (36) it is recommended that future research embodies the principles of co-design to address gaps within the mental health sector. WISE IDEA is actively working to involve those with lived experience in future study design and program implementation, including the involvement and consultation with lived experience researchers. Also, important to note, despite peer workers being involved in the co-facilitation of EYM, they were not allocated to the groups that the study participants took part in. This was due to the nature of EYM facilitator availability and allocation at that time. The co-facilitation of EYM by peer workers could have potential learnings for the Lived Experience workforce and should be further examined in future qualitative research.

Further qualitative investigations, acknowledging the limitations of the study such as the inclusion of peer workers as co-facilitators, larger sample sizes and a longitudinal design to further understand and explore the relationship between EYM and vocational outcomes are recommended.

### 4.3 Conclusion

The findings from this study enrich previous quantitative analysis by Miles et al. (21), providing a qualitative voice to further explore and understand the value of EYM. The appraisals from participants provide pivotal insight into subjective experiences, not readily available throughout quantitative outcomes.

Ultimately, findings of this study reinforce the necessity of a flexible approach to program delivery, the value of the therapeutic relationship and importance of a ‘just-right challenge’ to ensure positive participatory outcomes and fulfillment of vocational goals. The findings reflect the results of prior qualitative studies undertaken in CR, specifically providing a qualitative voice for the lived experience of the Employ Your Mind program.

## Data Availability

The raw data supporting the conclusions of this article will be made available by the authors, without undue reservation.
